# An interneuron progenitor maintains neurogenic potential *in vivo* and differentiates into GABAergic interneurons after transplantation in the postnatal rat brain

**DOI:** 10.1038/srep19003

**Published:** 2016-01-11

**Authors:** Qi Wang, Peiwei Hong, Hui Gao, Yuntian Chen, Qi Yang, Mei Jiang, Hedong Li

**Affiliations:** 1 West China Developmental & Stem Cell Institute, Department of Obstetric & Gynecologic and Pediatric, Key Laboratory of Obstetric & Gynecologic and Pediatric Diseases and Birth Defects, Ministry of Education, West China Second University Hospital, Sichuan University, Chengdu 610041, P.R. China; 2 Department of Neurology, West China Hospital, Sichuan University, Chengdu 610041, P.R. China; 3 Department of Urology, West China Hospital, Sichuan University, Chengdu 610041, P.R. China

## Abstract

Dysfunction of cortical GABAergic interneurons are involved in numerous neurological disorders including epilepsy, schizophrenia and autism; and replenishment of these cells by transplantation strategy has proven to be a feasible and effective method to help revert the symptoms in several animal models. To develop methodology of generating transplantable GABAergic interneurons for therapy, we previously reported the isolation of a v-myc-induced GABAergic interneuron progenitor clone GE6 from embryonic ganglionic eminence (GE). These cells can proliferate and form functional inhibitory synapses in culture. Here, we tested their differentiation behavior *in vivo* by transplanting them into the postnatal rat forebrain. We found that GE6 cells migrate extensively in the neonatal forebrain and differentiate into both neurons and glia, but preferentially into neurons when compared with a sister progenitor clone CTX8. The neurogenic potential of GE6 cells is also maintained after transplantation into a non-permissive environment such as adult cortex or when treated with inflammatory cytokine in culture. The GE6-derived neurons were able to mature *in vivo* as GABAergic interneurons expressing GABAergic, not glutamatergic, presynaptic puncta. Finally, we propose that v-myc-induced human interneuron progenitor clones could be an alternative cell source of transplantable GABAergic interneurons for treating related neurological diseases in future clinic.

GABAergic cortical interneurons serve as the major inhibitory neurons that form appropriate connections with excitatory projection neurons in the complex and highly ordered neuronal circuitry of the mammalian cerebral cortex^1^,^2^. Unlike locally produced projection neurons, GABAergic interneurons have to migrate a long distance to the cortex from their birth place, ganglionic eminences (GE) of the ventral telecephalon, during embryonic stages^3^,^4^. In the cerebral cortex, GABAergic interneurons help modulate firing patterns of projection neurons through forming inhibitory synapses onto different parts of the cellular regions in order to maintain balance of inhibition and excitation in the cortical neuronal circuitry^5^,^6^. Dysfunction of GABAergic interneurons in disrupting this balance due to either genetic mutations or injury is thought to involve in a panel of neurological disorders including epilepsy, schizophrenia and autism^7^,^8^.

The therapeutic potential of GABAergic interneurons in treating these diseases has been highly recognized recently since numerous groups demonstrated successful cases by transplantation of medial GE (MGE)-derived interneuron precursors^9^,^10^. A notable characteristic of these cells is their ability to migrate in the neonatal and adult brain expanding their potential in affecting a wide area of diseased brain. This migratory capacity is thought to be intrinsically determined and related to the native developmental profile of these cells during embryonic stages^11^. GABAergic interneuron transplantation has been shown to benefit in animal’s behaviors in numerous disease models including epilepsy^12^,^13^,^14^, schizophrenia^15^, Parkinson’s^16^ and spinal cord injury^17^. In most cases, functional GABAergic interneuron integration seems to be required to facilitate recovery, although other mechanisms such as increase in cortical plasticity by these transplanted cells are also proposed^18^.

Given the rapid advance in transplantation of GABAergic interneuron precursor for treating neurological diseases in animal models, renewable sources of such GABAergic interneurons are in high demand. Primary MGE-derived cells are unlike to be a feasible source in a future clinical setting. Derivation of GABAergic interneuron from ESCs or iPSC by genetic^19^ and culturing induction^20^,^21^,^22^,^23^,^24^ has been attempted but the results are not satisfactory and efficiency is low^21^. In addition, functional improvement by transplantation of these derived interneurons does not always meet expectation^25^,^26^,^27^. Therefore, alternative sources of these cells are clearly needed. Generation of neural stem cell (NSC) clones by Myc-transduction has been developed decades ago, and therapeutical potentials of these clones have been extensively demonstrated^28^,^29^.

Our previous report has demonstrated that GE6 cells proliferate rapidly in culture in the presence of FGF2 and differentiate into primarily neurons with little astroglia upon FGF2 withdrawal^30^. In the current study, we aim to determine if this distinct neurogenic potential of GE6 still holds after transplantation into the postnatal brain. Furthermore, we explore to optimize the pretreatment of GE6 cells before transplantation in order to facilitate future transplantation of similar human cells in a clinical setting. We found that transplanted GE6 cells exhibit robust migratory property, like their *in vivo* counterpart, and that these cells show some differentiation plasticity, but still maintain higher neurogenic potential when compared with transplanted CTX8 multipotential NSC clone. In addition, a simple predifferentiation treatment of GE6 helps improve survival of grafted rats and differentiation of GE6 cells in the postnatal cerebral cortex.

## Results

### Transplanted GE6 cells show robust migratory property and morphological differentiation in different regions of the postnatal forebrain

**Figure 1:**
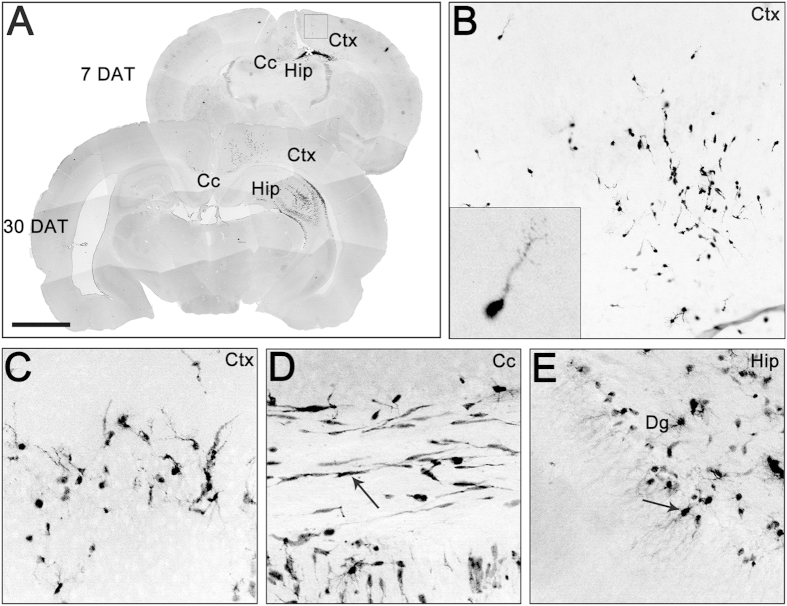
Migration and morphological differentiation of GE6 cells following transplantation into the neonatal rat forebrain. (**A**) Stacked images of inverted GFP signal showing distributions of GE6 cells at 7 and 30 days after transplantation (DAT) into the neonatal rat forebrain. The asterisk indicates the putative cell injection site in the 7-DAT forebrain. (**B**) Enlarged image of the boxed region in the 7-DAT forebrain in (**A**). The insert shows a typical migratory morphology of transplanted GE6 cells in the Ctx. Differentiated cell morphologies of transplanted GE6 cells are shown in the Ctx (**C**), Cc (**D**) and Hip (**E**) of the 30-DAT forebrain. Typical region-specific morphologies are indicated by arrows in (**D**) and (**E**). Ctx, cortex; Cc, corpus callosum; Hip, hippocampus; Dg, dentate gyrus. Scale bar, 500 μm in (**A**), 200 μm in (**B**), 100 μm in (**C**–**E**).

We previously reported a panel of neural progenitor clones derived from an E14.5 GFP rat forebrain using v-myc transduction^30^. Among them, one such clone GE6, isolated from the GE region, displays properties of GABAergic interneuron progenitor preferentially giving rise to interneurons with the capacity of forming functional synaptic connections with primary hippocampal neurons and themselves in culture^30^. To evaluate the capability of GE6 cells to replenish interneurons, we transplanted them into the neonatal rat forebrain to examine their behavior *in vivo* by a protocol modified from a previous report^31^. A single point injection of 10,000 cells was made unilaterally aiming for one side of the subventricular zone (SVZ) of the P1-P3 rat pups. Like their *in vivo* counterpart (the MGE cells), GE6 cells dispersed nicely from the injection site and migrated into cortex (Ctx), corpus callosum (Cc) and hippocampus (Hip) at 7 days after transplantation (DAT) (Fig. 1A). Many GE6 cells with the migratory morphology, i.e. a tear drop-shaped cell body and a leading process, were observed in the Ctx at 7 DAT (Fig. 1B, insert). At 30 DAT, more GE6 cells were found to migrate out of the injection site and into the forebrain regions, and some cells were even found on the contralateral side of the brain, likely migrated through the Cc (Fig. 1A). Higher magnification images revealed characteristic morphologies of GE6 cells in different regions of the forebrain. For example, GE6 cells with a mixed morphology in the Ctx suggest their differentiation into distinct cell types (Fig. 1C). GE6 cells in the Cc exhibit a long bipolar morphology suggesting their differentiation into the oligodendrocyte lineage (Fig. 1D, arrow), while some GE6 cells in the dentate gyrus (Dg) of the Hip take on the morphology of putative granule neurons, a small cell body with long and highly branched processes (Fig. 1E, arrow). Therefore, our analysis by GFP microscopy indicates that GE6 cells maintain their native migratory property and disperse nicely in the postnatal forebrain upon transplantation, and that, morphologically, these cells differentiate into region-specific cell types.

### Transplanted GE6 cells respond to local cues and differentiate into distinct cell types in the postnatal forebrain

**Figure 2:**
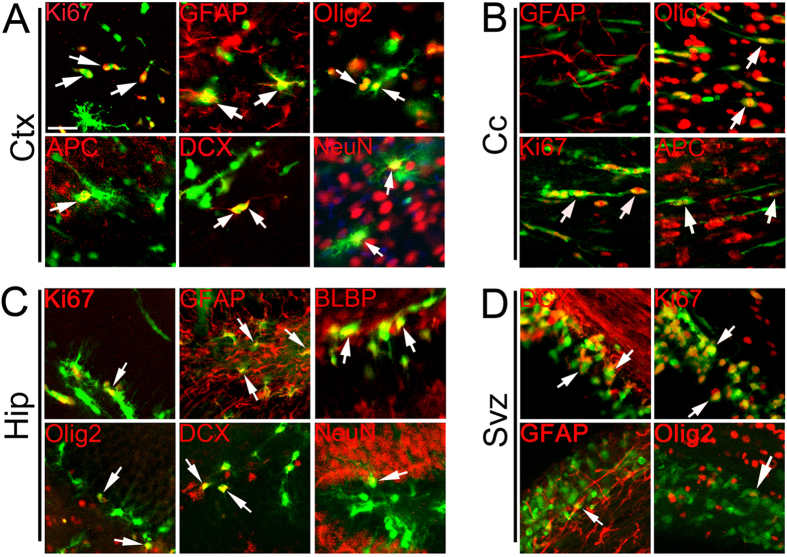
Immunological characterization of GE6 cell differentiation in different regions of the forebrain at 30 DAT following transplantation into the neonatal rat forebrain. Immunostainings with cell type-specific markers show differentiation of transplanted GE6 cells in the Ctx (**A**), Cc (**B**), Hip (**C**) and SVZ (**D**) of the forebrain at 30 DAT. GE6 cells are indicated by GFP signal. Arrows indicates double-positive cells. Ctx, cortex; Cc, corpus callosum; Hip, hippocampus; SVZ, sub-ventricular zone. Scale bar, 20 μm.

Next, we examined further the differentiation of GE6 cells in different forebrain regions by immunostaining. In the Ctx, many GE6 cells were found to express the proliferation marker Ki67 indicating they were actively proliferating, and these cells usually had simple cellular morphology (Fig. 2A). Some cells also expressed astrocytic marker GFAP, while others express oligodendrocyte lineage markers Olig2 and APC. Neuronal differentiation of GE6 cells was also observed by their expression of Doublecortin (DCX) (young neurons) and NeuN (mature neurons) (Fig. 2A). Thus, transplanted GE6 cells differentiated into major neural cell types of the central nervous system (CNS) while maintaining a portion as dividing progenitors. However, in the Cc, consistent with their elongated cell morphology (Fig. 1D), many GE6 cells were found to express Ki67, Olig2 and APC indicating a predominant differentiation process towards oligodendrocytes, but not astrocytes (GFAP^+^) (Fig. 2B). Very rarely were DCX^+^ or NeuN^+^ neuronal GE6 cells found in this region (data not shown). Similar to the Ctx, the Hip contained GE6 cells that were differentiated into distinct cell types including astrocytes (GFAP^+^) and neurons (DCX^+^ and NeuN^+^), in addition to undifferentiated progenitors (Ki67^+^) and radial glia (BLBP^+^) (Fig. 2C). Some transplanted GE6 cells were also observed in the SVZ where they mostly expressed Ki67 and DCX suggesting their involvement in adult neurogenesis, while very few of these cells expressed GFAP and Olig2 in this region (Fig. 2D). Together, these results indicate that GE6 cells retain some differentiation plasticity, giving rise to distinct cell types in a region-specific manner.

### GE6 cells maintain their intrinsic neurogenic potential upon transplantation into the neonatal cerebral cortex

**Figure 3:**
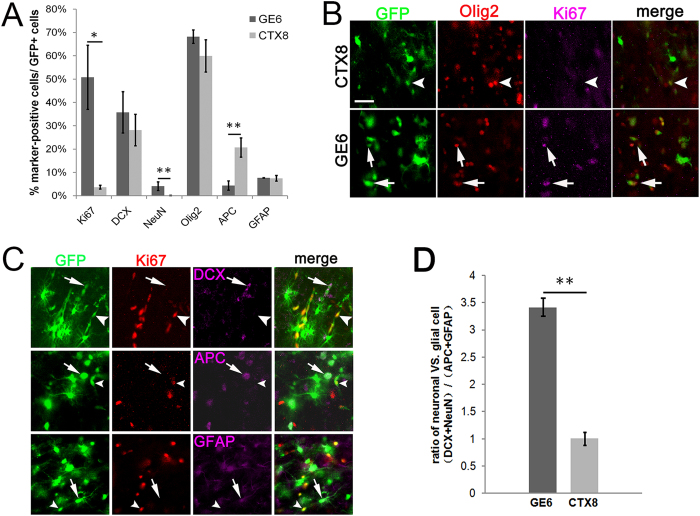
Preferential neurogenic potential of GE6 cells in the Ctx at 30 DAT following transplantation into the neonatal rat forebrain. (**A**) Comparison of cell type marker-positive cells between transplanted GE6 and CTX8 cells in the Ctx. (n = 3 for both GE6 and CTX8). (**B**) Co-immunosatinings showing that, while most Olig2^+^ CTX8 cells are Ki67^−^ (arrowheads), many Olig2^+^ GE6 cells are also Ki67^+^ (arrows) in the Ctx. (**C**) Co-immunostainings showing a non-overlapping expression of Ki67 and cell type-specific markers among transplanted GE6 cells in the Ctx. Arrows indicate cell type marker-positive GE6 cells, while arrowheads indicate Ki67^+^ GE6 cells. (**D**) Comparison of differentiation potential to neuronal vs. glial cell types between transplanted GE6 and CTX8 cells in the Ctx. Scale bar, 20 μm. *P < 0.05; **P < 0.01 by Student’s *t*-test.

We previously reported that neural progenitor clone GE6 differentiates into primarily interneurons and few astroglia in culture, while a sister clone CTX8 differentiates into both neurons and astroglia^30^. Based on these observations, we proposed that the cell fate of GE6 cells is intrinsically determined or biased by their expression of a unique combination of transcription factors including DLXs so that the environmental cues may play little roles in directing differentiation of these cells. Thus, transplantation of GE6 cells to replenish interneurons may be advantageous in disease or injury models where local environments are usually non-permissive for neuronal differentiation. To determine if the intrinsic neurogenic potential of GE6 holds true *in vivo*, at a first step, we transplanted GE6 cells into the neonatal forebrain and compared differentiation of these cells with that of the multipotential progenitor clone CTX8 in the Ctx where permissive cues exist (Figs 1C, 2A). We found that about 50% of transplanted GE6 cells in the Ctx were still proliferating progenitors (Ki67^+^) at 30 DAT while this percentage was less than 5% for CTX8 (Fig. 3A). This remarkable difference in cell proliferation between these two sister progenitor clones may represent their intrinsically distinct nature and correlate with the higher v-myc expression in GE6 comparing to CTX8^30^. For neuronal differentiation, the two clones showed a similar percentage of DCX^+^ cells among transplanted cells, but GE6 displayed a significant higher percentage of NeuN^+^ cells than CTX8, which had essentially none (Fig. 3A). The percentages of cells expressing astroglial marker GFAP and oligodendrocyte marker Olig2 showed no difference, whereas the mature oligodendrocyte marker APC was higher in percentage in CTX8 than in GE6. A closer examination revealed that most Olig2^+^ GE6 cells also expressed Ki67 (Fig. 3B, arrows) but Olig2^+^ CTX8 cells did not (Fig. 3B, arrowhead). This suggests that Olig2^+^ GE6 cells in the Ctx represent mostly undifferentiated cells; whereas Olig2^+^ CTX8 cells are in fact oligodendrocyte lineage cells including precursors and APC^+^ mature oligodendrocytes. To compare the differentiation potential of GE6 and CTX8 directly, we first confirmed that Ki67 did not co-localize with cell type markers DCX, APC, GFAP (Fig. 3C) and NeuN (data not shown), and then examined only differentiated cells by comparing the ratio of neuronal (DCX + NeuN) vs. glial (APC + GFAP) differentiation between GE6 and CTX8. Clearly, this ratio of differentiation is significantly, and 3-fold, higher for GE6 than CTX8 (Fig. 3D), indicating a preferential neurogenic potential of GE6 cells when transplanted into the Ctx of the neonatal forebrain. Therefore, these results suggest that the intrinsic neurogenic potential of GE6 cells is maintained in a relatively permissive *in vivo* environment.

### GE6 cells maintain their intrinsic neurogenic potential upon transplantation into the adult cerebral cortex

**Figure 4:**
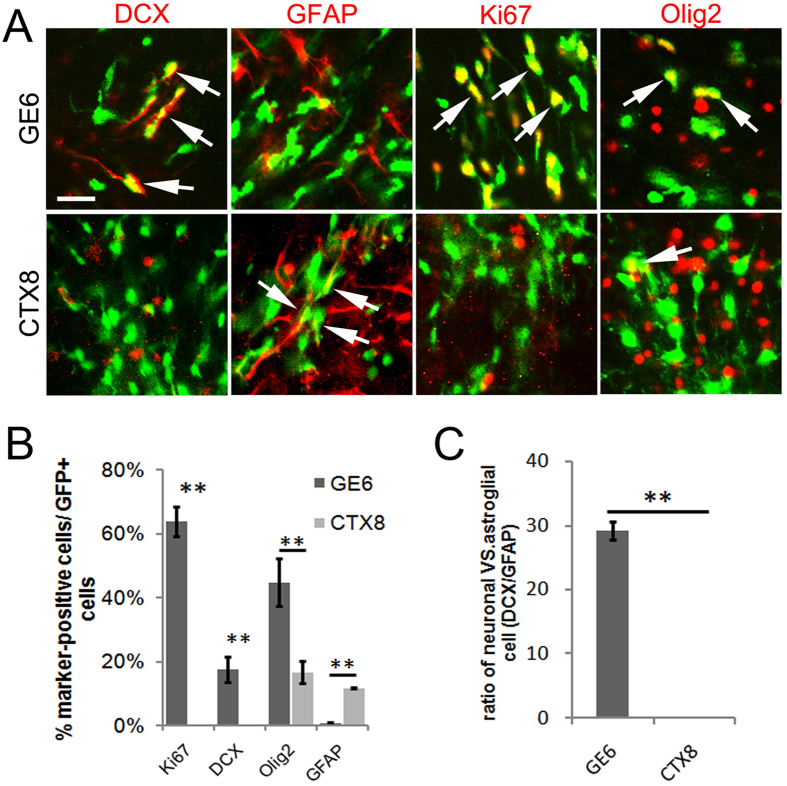
Preferential neurogenic potential of GE6 cells in the Ctx at 15 DAT following transplantation into the adult rat forebrain. (**A**) Immunological characterization of transplanted GE6 and CTX8 cells (indicated by GFP signal) in the Ctx. Arrows indicate double-positive cells. (**B**) Comparison of cell type marker-positive cells between transplanted GE6 and CTX8 cells in the Ctx (n = 3 for both GE6 and CTX8). (**C**) Comparison of differentiation potential to neuronal vs. astroglial cell types between transplanted GE6 and CTX8 cells in the Ctx. Scale bar, 20 μm. *P < 0.05; **P < 0.01 by Student’s *t*-test.

To challenge the neurogenic potential of GE6 in a non-permissive condition, we transplanted these cells into the adult rat brain where neurogenesis is mostly completed. We compared differentiation of GE6 cells in the adult cortex with that of CTX8 at 15 DAT, and found that while more than 60% of transplanted GE6 cells remained as proliferating progenitors (Ki67^+^), a significant proportion of these cells took on neuronal phenotype by expressing DCX (Fig. 4A,B). In contrast, transplanted CTX8 cells showed almost none of Ki67^+^ or DCX^+^ cells, but a significantly higher percentage of GFAP^+^ astrocytes than GE6, which has none. Similar to our observation in the neonatal transplantation, we confirmed that Olig2^+^ transplanted cells in the adult Ctx represented mostly Ki67^+^ proliferating cells in GE6 but oligodendrocyte lineage cells in CTX8 (data not shown). In addition, a similar ratio of neuronal vs. astroglial cells was calculated and, again, revealed a drastic neurogenic preference of differentiation for GE6 cells (Fig. 4C). Of note, this 15-day transplantation is insufficient for cell type maturation as we did not observe any mature neurons (NeuN^+^) or oligodendrocytes (APC^+^), and yet it allowed examination on cell fate determination of transplanted cells and initiation of their differentiation process. Thus, these results showed that GE6 cells maintained their neurogenic potential when transplanted into the adult cerebral cortex.

### GE6 cells maintain their intrinsic neurogenic potential when treated with inflammatory cytokine leukemia inhibitory factor (LIF) in culture

**Figure 5:**
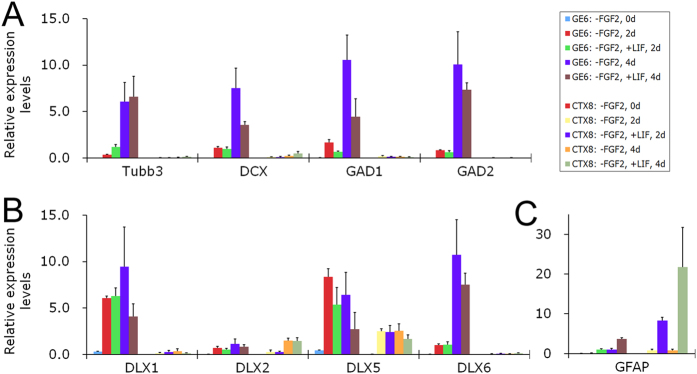
Gene expression analysis of neural progenitor clones (GE6 and CTX8) during differentiation in culture upon LIF treatment. Neural progenitor clones (GE6 and CTX8) were allowed to differentiate in culture by FGF2 withdrawal (-FGF2). These cells were then treated with inflammatory cytokine LIF (50 ng/ml) for 2 or 4 days upon differentiation; and gene expression was analyzed by qRT-PCR. (**A**) Neuronal and GABAergic interneuronal genes; (**B**) DLX transcription factors; (**C**) Astrocyte marker gene GFAP. The individual value represents the average of three replicates with a standard deviation.

To further challenge the neurogenic potential of GE6 cells in an injury-like environment, we wanted to test the differentiation of GE6 progenitor cells in lipopolysaccharide (LPS)-induced *in vitro* injury model as we previously described^32^. After treatment of LPS (100 ng/ml) for 2 or 4 days in culture, we first examined the gene expression of inflammatory factors such as *IL-2, IL-6, LIF, TNF-alpha,* and *INF-gamma* by quantitative reverse transcriptase PCR (qRT-PCR). To our surprise, we did not observe a significant difference in the expression level of any of these inflammatory factor genes in either GE6 or CTX8 cultures (data not shown). This result is in great contrast to what we have observed in LPS-treated primary mouse astrocyte cultures^32^ and suggests that these neural progenitors are insensitive to LPS treatment. LPS-induced response is mainly mediated by microglia and reactive astrocytes^33^; and the lack of responsiveness to LPS in neural progenitor cells is probably due to the absence of inflammatory factor producers in their cultures. We then directly applied LIF (50 ng/ml), one of the major inflammatory cytokines induced by LPS^32^, to neural progenitor cultures and examined their differentiation by qRT-PCR. During the time-course differentiation, LIF treatment did not inhibit the expression of neuronal gene Tubb3 in GE6 (Fig. 5A). Although the expression of DCX, a marker of young neurons, was somewhat decreased upon LIF treatment in GE6 at day 4, the expressions of these two neuronal genes were much higher in GE6 than in CTX8 in general (Fig. 5A). We also examined the expression of GABAergic interneuron markers GAD1 and GAD2. Similar result was observed that LIF treatment moderately reduced their expressions in GE6 cells at day 4, but their expressions in GE6 were still much higher than those in CTX8, which were essentially none (Fig. 5A). DLX transcription factors play critical roles in the development of GABAergic interneurons^34^. We found that LIF treatment had a moderate effect on the expression of these genes during GE6 differentiation, but again GE6 cells had substantially higher DLX expression (especially DLX1, DLX5, and DLX6) than CTX8 cells even in the presence of LIF (Fig. 5B). These results suggest that persistent DLX expression of GE6 cells in an injury-like environment (LIF-treatment) intrinsically instructs these cells to take on interneuronal cell fate upon differentiation. On the other hand, astrocytic cell fate was slightly promoted by LIF during GE6 differentiation suggesting some plasticity of these cells in differentiation potential that can be affected by environmental cues. In great contrast, LIF induced dramatic astrocytic differentiation in CTX8 as indicated by a huge increase in GFAP expression level (Fig. 5C). This result is in agreement with the observation that multipotent neural stem cells mostly differentiate into astrocyte lineage after transplantation into adult or injured CNS^35^. In sum, this gene expression analysis indicates that intrinsic neurogenic potential of GE6 cells, probably governed by persistent expression of key transcription factors such as DLXs, can be maintained even in an injury-like condition, while multipotent neural progenitor CTX8 is more easily affected by environmental cues and mostly differentiates into astrocytes when stimulated by injury-released, glia-inducing cytokines.

### Predifferentiation of GE6 cells before transplantation improves survival of grafted rats and shows increased overall differentiation in the postnatal cerebral cortex

**Figure 6:**
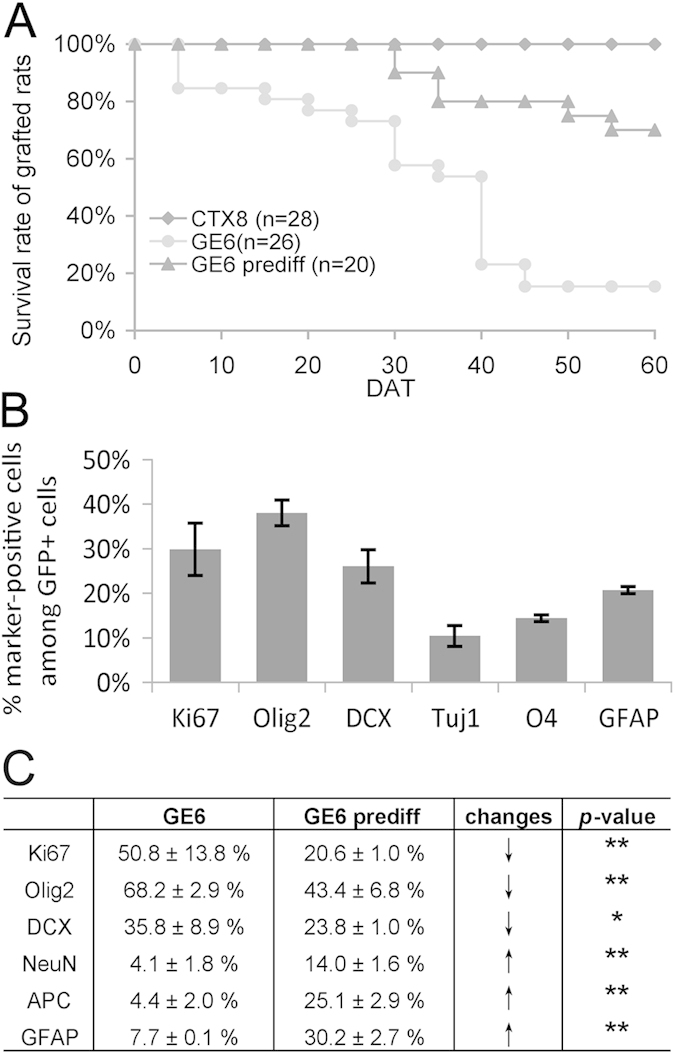
Transplantation of predifferentiated GE6 cells into the neonatal rat forebrain enhances their overall differentiation in the Ctx at 30 DAT and improves survival of transplanted rats. (**A**) Survival analysis of rats transplanted with CTX8, GE6 and predifferentiated GE6 (GE6 prediff). (**B**) Quantification of cell type-specific marker expression in GE6 cells after predifferentiation for 3 days *in vitro*. (**C**) Comparison of cell type marker-positive cells between GE6 and GE6 prediff upon transplantation in the Ctx at 30 DAT (n = 3 for both GE6 and GE6 prediff). *P < 0.05; **P < 0.01 by Student’s *t*-test.

Relative to CTX8, much higher percentage of Ki67^+^ cells in transplanted GE6 even at 30 DAT in the Ctx may help explain a more frequent occurrence of cell mass formation in GE6-transplanted rats. This cell mass formation could be lethal as we observed that the survival rate of GE6-grafted rats was below 60% at 30 DAT and down to 15% at 60 DAT, while essentially no death was seen in CTX8-grafted rats within 60 DAT (Fig. 6A). The cell masses contained mostly Ki67^+^ and cleaved caspase-3^+^ cells with a small number of DCX^+^ neurons indicating that proliferation and cell death seem to occur simultaneously (data not shown). In addition, cell masses were often found to form near the SVZ where one of the neural stem cell niches has been described^36^,^37^, and their sizes and speed of formation seemed to correlate with the number of GE6 cells transplanted (unpublished observations). To increase the survival rate of GE6-transplanted rats, we adopted a predifferentiation protocol that has been shown to speed up the process of functional maturation and synaptic formation of GE6 cells in culture^30^. Indeed, this predifferentiation protocol of GE6 before transplantation helped improve survival rate of grafted rats up to 70% at 60 DAT allowing observation of neuronal maturation of transplanted GE6 cells *in vivo* (Fig. 6A). We characterized the predifferentiated GE6 cultures and found that proliferating cells (Ki67^+^ and Olig2^+^) were reduced to 30-40%, and that neuronal differentiation had already occurred shown by DCX^+^ and TuJ1^+^ neurons as well as glial differentiation shown by O4 and GFAP immunoreactivity (Fig. 6B). Consistently, the predifferentiation significantly reduced percentage of Ki67^+^ GE6 cells as well as Olig2^+^ cells in the Ctx at 30 DAT (Fig. 6C). Although the percentage of DCX^+^ young neurons were also reduced, the percentage of NeuN^+^ mature neurons were increased more than 3-fold suggesting an improved neuronal maturation of transplanted GE6 cells *in vivo* by the predifferentiation. Surprisingly, percentages of glial marker (APC and GFAP)-positive cells were also significantly increased suggesting that the predifferentiation protocol did not bias neuronal differentiation at the expanse of glial differentiation (Fig. 6C). Taken together, the 3-day predifferentiation protocol help improve the survival rate of GE6-grafted rats probably by reducing cell proliferation and promoting overall differentiation of these cells before transplantation.

### Morphological and biochemical maturation of transplanted GE6 cells as GABAergic interneurons in the postnatal forebrain

**Figure 7:**
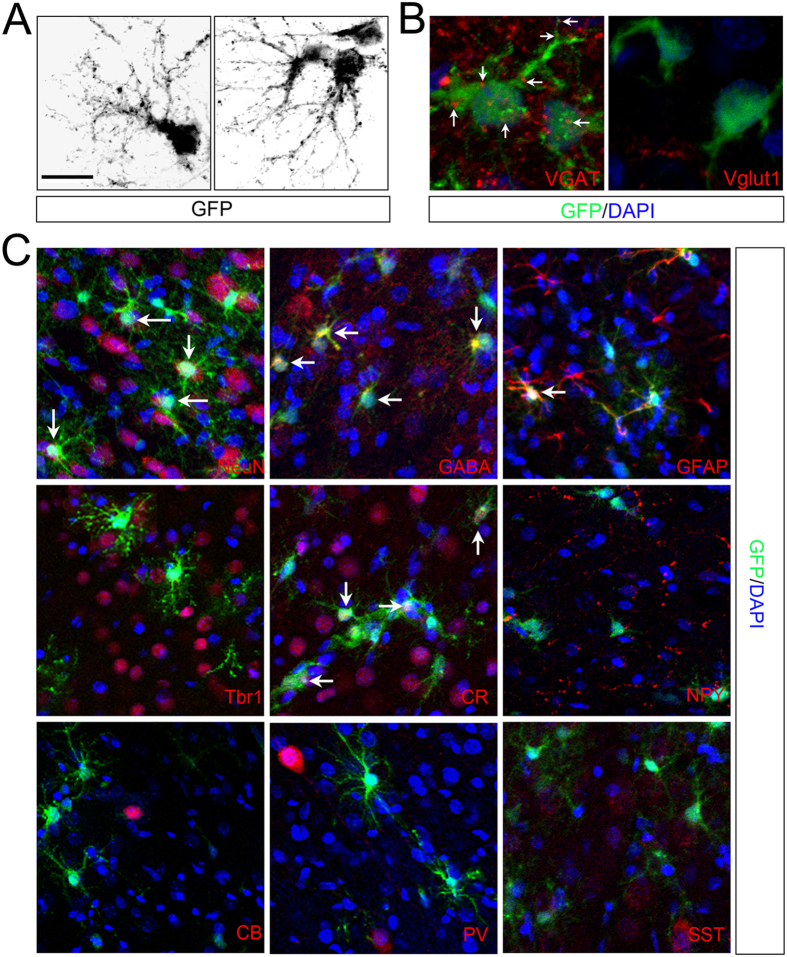
Predifferentiated GE6 cells transplanted into the neonatal rat forebrain display mature neuronal phenotypes in the Ctx at 120 DAT. (**A**) complex neuronal morphology of predifferentiated GE6 cells revealed by inverted GFP signal. (**B**) Immunostaining of subtype-specific presynaptic markers (VGAT and Vglut1). Arrows indicate positive puncta around transplanted GE6 cells. (**C**) Immunostaining of cell type and subtype specific markers to show identity of transplanted GE6 cells in the Ctx. Arrows indicate double-positive cells. DAPI is used to label nuclei. Scale bar, 20 μm in (**A**), 10 μm in (**B)**, 40 μm in (**C)**.

Increased animal survival by transplantation of predifferentiated GE6 cells allowed us examine neuronal maturation of this v-Myc transduced neural progenitor clone *in vivo*. In the cerebral cortex of 60-DAT animals, GFP^+^ GE6 cells with complex neuronal morphology are more often seen than in 30-DAT animals. These cells typically have enhanced dendritic processes with spine-like structures suggesting their synapse formation in the local circuitry (Fig. 7A). Staining with pre-synaptic markers shows that VGAT^+^ puncta can be observed around the cell body and dendrites, but Vglut1^+^ puncta are rarely seen to co-localize with GFP (Fig. 7B). In addition, unlike Tbr1^+^ cortical projection neurons, GE6-derived neurons usually have a smaller cell body and no axon, which resembles cortical GABAergic interneurons (Fig. 7C). Among the GE6 cells with complex neuronal morphology, more than half are NeuN^+^ and GABA^+^, few are GFAP^+^. These cells do not show proliferation marker expression, and show no colocalizaion with projection neuron marker Tbr1 (Fig. 7C). These data indicate that, upon transplantation into the postnatal cerebral cortex, GE6 cells mainly acquire GABAergic interneuron phenotype and integrate into the host cortical tissue. Previously, we showed GE6 cells give rise to GABAergic interneuron subtypes in culture after 6-day differentiation^30^. Neuropeptide Y (NPY), Somatostatin (SST) and Parvalbumin (PV) are the major interneuron subtypes that GE6 cells differentiate into in culture. Here we also examined these subtype marker expression in transplanted GE6 cells and used it as a maturation criteria. In contrast to *in vitro* results, transplanted GE6 cells mainly express Calretinin (CR) (~43%) and few NPY, but no other subtype markers (Fig. 7C). The distinct interneuron subtype specification of GE6 cells between *in vitro* and *in vivo* indicates plasticity of these cells in giving rise to different subtypes of GABAergic interneurons, which can be manipulated to better suit particular therapeutic purposes.

## Discussion

During CNS development, multipotential neural stem cells become diversified in a spatiotemporal fashion and progressively restricted in their choices of cell types that they can give rise to. These cell-fate-restricted progenitors maintain proliferation ability and generate specific neural cell types in a precisely controlled manner before exhausted at late stages of development^28^,^29^. Our success in isolating a variety of neural progenitors from different regions of the E14.5 rat forebrain by v-myc immortalization strongly supports the presence of these diversified neural progenitors^30^. In addition, the fact that the unique properties of these progenitors can be sustained in culture for many passages encourages the usage of these progenitors in transplantation for a potential cell-based therapy. Isolation of progenitors with distinct differentiation potentials (i.e. neurogenic, gliogenic or multipotential) signifies their intrinsic natures (gene expression profiles) that help maintain their properties at least for a certain period of time. For example, Dlx-expressing interneuronal progenitor GE6 was able to gives rise to GABAergic interneurons in culture after at least 30 passages (data not shown). In this study, we report that the neurogenic potential of GE6 maintains *in vivo* compared with a sister clone CTX8. However, we did observe some plasticity of transplanted GE6 cells in that differentiation of cell types differ between regions where cells were transplanted. These include cellular morphology, degree of differentiation and maturation, and expression of cell type-specific markers. Thus, region-specific factors may also play a role in the behavior of transplanted cells. The environment in the adult brain favors glial differentiation of transplanted NSC^38^. However, our result that much more transplanted GE6 cells differentiate into DCX^+^ neurons than CTX8 within two weeks in the adult cortex (Fig. 4) or when challenged by inflammatory cytokine *in vitro*, shows feasibility that natural intrinsic factors may overcome a non-permissive extrinsic environment to direct cell differentiation. It will be exciting to test if the neurogenic property of GE6 cells retains in a diseased or injury CNS where neuronal replacement is needed.

Along with other genes, v-myc has been used as an immortalizing gene for decades, and v-myc transduced NSC clones have been reported previously^29^. However, GABAergic interneuronal progenitor clones by v-myc have not been reported. A central aim of this study is to test the feasibility of such progenitor clone as a cell source of transplantable GABAergic interneurons. Our results are promising in that GE6 cells are able to migrate extensively in the postnatal brain upon transplantation and differentiate into GABAergic interneurons in the Ctx with expression of mature interneuron and synaptic markers. V-myc expression in this interneuron progenitor drastically reduces upon differentiation (both *in vitro* and *in vivo*) and does not seem to interfere with its differentiation potential^30^. Although the neuronal maturation of transplanted GE6 cells as determined by appearance of synaptic structure and subtype-specific markers was achieved in the Ctx, the timing of maturation process may be altered by v-myc expression. The MGE-derived primary cells were able to mature in 30 days after transplantation in the neonatal forebrain^12^. However, v-myc-induced GE6 cells take at least 60 days to show maturation phenotypes (Fig. 7). This timing difference may be due to a potential anti-differentiation function of v-myc gene in GE6 cells or the fact that cells derived from MGE contain a large number of interneurons and supersede GE6 cells in the differentiation process even before transplantation. Nevertheless, the v-myc induced interneuronal progenitors such as GE6 could serve as a potential source of transplantable GABAergic interneurons for cell-based therapy. The unique developmental origin of GABAergic interneurons in both rodents^11^ and human^39^,^40^ facilitates isolation of these progenitors and therapeutic potential of this particular neuronal cell type in related neurological disorders.

Although v-myc has been regarded as a non-transforming gene and previous transplantation studies using v-myc induced neural progenitors show no signs of tumorigenecity^29^, we did observe cell mass formation after transplantation of GE6 cells especially transplantation of undifferentiated GE6 cells. This correlates with higher v-myc expression in GE6 compared with other clones such as CTX8. Transplantation of GE6 cells that have been predifferentiated in culture greatly reduces cell mass formation and increases survival of grafted animals (Fig. 6). Therefore, for therapeutic application of GE6-like interneuronal progenitors, a pretreatment to slow down cell proliferation and initiate differentiation process is likely required. Furthermore, a selection or enrichment of postmitotic neurons, for example, by FACS using neuronal cell surface markers, could potentially increase the efficacy of neuronal replacement and further reduce cell mass formation.

Alternative immortalizing methods including tetracycline-controllable myc expression system could further increase safety of these cells after transplantation. Although reduced v-myc expression level does not seem to interfere with interneuronal differentiation and maturation^29^,^30^, residual v-myc level in the mature neuronal cells may affect their physiological functions and potentially tumorigenic in longer term. Therefore, a tighter control on v-myc expression would eliminate these potential problems. In an ideal scenario, myc expression is turned on when target cells are expanded into a large quantity that is required for transplantation and turned off when they differentiate into mature neuronal cell types after transplanted into the host CNS. This type of approach is currently being investigated in our laboratory.

In conclusion, this study deals with the potential source of therapeutic GABAergic interneurons for transplantation. We tested feasibility of v-myc expanded interneuron progenitors as cell source by implanting progenitors and predifferentiated cells into the postnatal brain. We showed *in vivo* differentiation and maturation of these cells in different regions of the brain, although further optimization could be done to increase the efficacy of differentiation and decrease the potential tumorigenecity.

## Methods

### Animal uses

Sprague-Dawley rats were purchased from Chengdu Dossy Experimental Animals Co., Ltd (Chengdu, P.R. China). All animal use and studies were approved by the Institutional Animal Care and Use Committee of West China 2nd Hospital, Developmental & Stem Cell Institute, Sichuan University, Chengdu, P.R. China. All procedures were carried out in accordance with the approved guidelines.

### Cell culturing, differentiation, and factor treatment

GE6 and CTX8 cells were cultured in a serum-free medium containing FGF2 as described^30^. In some experiments, GE6 cells were differentiated in a differentiation medium containing no FGF2 but 0.5% fetal bovine serum (FBS) for 3 days. These partially differentiated cultures were harvested for transplantation and referred to as “predifferentiated GE6”. For factor treatments, GE6 and CTX8 cells were allowed to differentiate in the absence of FGF2^30^, but in the presence of LPS (100 ng/ml) or LIF (50 ng/ml) for 2 or 4 days before harvest for total RNA.

### Total RNA extraction and qRT-PCR

Total RNA extraction and qRT-PCR analysis of gene expression were performed as previously described^41^. The relative gene expression levels were normalized to that of the housekeeping gene *GAPDH*. The primer sequences of the target genes are shown in Table 1.

### Transplantation procedure

For neonatal rat transplantation, P1-P3 rat pups were anesthetized on ice for about 5 minutes until no pedal reflex was observed, and this anesthesia was maintained on an ice-cold aluminum plate throughout the transplantation procedure. Dissociated cells (GE6 or CTX8) were concentrated to a high density (~5 × 10^5^/μl) and back-loaded into a glass pipette tip (~50 μm in inner-diameter) using a long-end Eppendorf micropipette tip. The glass pipette was then mounted onto a stereotaxic microprocessor (MC-4, TaiMeng Technology, Chengdu, P.R. China), and cell injection was made using an electronic air pump injector (Picospritzer^®^ III, Parker Hannifin Corp., New Jersey, USA). A single point injection with a total of 10,000 cells in a 20 nl volume was made on the left-side of the forebrain perpendicular to the skin surface (coordinate: 3.5 mm anterior, 1 mm lateral, 1.5 mm dorsal, aiming for the SVZ region). The injected pups were returned to their mothers after a 5-minute recovery on a warming pad and analyzed after1, 2, 3, 4 and 8 weeks.

For adult rat transplantation, 2-month-old rats were anesthetized using isoflurane (3% with oxygen). A sagittal incision of ~1 cm long was made on the midline of the scalp skin. An opening of 1.5 mm in diameter was made on the skull using an electrical dental drill (coordinate: 4.5 mm anterior, 2 mm lateral, 2 mm dorsal). A single point injection with a total of 10,000 cells in a 20 nl volume was made on the left-side of the forebrain perpendicular to the brain surface. The injected rats were analyzed after 2 weeks.

### Immunohistochemistry, immunocytochemistry and fluorescence microscopy

Forebrains were collected after intra-cardiac perfusion with phosphate buffered saline (PBS) followed by 4% paraformaldehyde (PFA) in PBS. The tissues were then coronally sectioned into 40 μm-thick section on a Vibratome and post-fixed in 4% PFA overnight at 4 °C. Tissue sections or fixed cell cultures were incubated with monoclonal antibodies against GFP (mouse, 1:500, Millipore), NeuN (mouse, 1:400, Millipore), GFAP (mouse, 1:500, Millipore), APC (mouse, 1:200, Millipore), GABA (mouse, 1:1000, Sigma), TuJ1 (mouse, 1:500, Covance), O4 (mouse, 1:100, Millipore), and CD11b (mouse, 1:500, Millipore); polyclonal antibodies against GFP (rabbit, 1:500, Millipore), BLBP (rabbit, 1:400, Abcam), Ki67 (rabbit, 1:500, Millipore), Olig2 (rabbit, 1:500, Millipore), GFAP (rabbit, 1:2000, Dako), DCX (guinea pig, 1:1000, Millipore), VGAT (rabbit, 1:500, Synaptic Systems), Vglut1 (rabbit, 1:500, Synaptic Systems), Tbr1 (rabbit, 1:400, Proteintech), PV (rabbit, 1:800, ImmunoStar), Calbindin (CB) (rabbit, 1:500, ImmunoStar), CR (rabbit, 1:500, ImmunoStar), NPY (rabbit, 1:500, ImmunoStar), and SST (rabbit, 1:500, ImmunoStar), followed by appropriate species-specific secondary antibodies (Molecular Probes). DAPI (10ug/ml, Sigma) was included in the secondary antibody incubations to label nuclei. The sections or fixed cultured cells were then mounted in mounting medium (Zhong Shan Golden Bridge Biotech, P.R. China) and analyzed by conventional or confocol fluorescence microscopy.

### Statistical analysis

All quantifications on sections were done with tissues from at least three animals. All data were presented as means ± standard deviation. Statistical analysis was performed in Microsoft Excel using Student’s *t*-test.

## Additional Information

**How to cite this article**: Wang, Q. *et al.* An interneuron progenitor maintains neurogenic potential in vivo and differentiates into GABAergic interneurons after transplantation in the postnatal rat brain. *Sci. Rep.* **6**, 19003; doi: 10.1038/srep19003 (2016).

## Figures and Tables

**Table 1:** Primers used for qRT-PCR.

Gene Name	Forward	Reverse
*GAPDH*	tgagatcaacgtgttccagtg	accagatgaaatgtgcccc
*IL-2*	tgttgctggacttacaggtg	agctccgagttcattttccag
*IL-6*	aagccagagtcattcagagc	gtccttagccactccttctg
*LIF*	ttcccatcacccctgtaaatg	aatggttccccttgagctg
*TNF-alpha*	catccgttctctacccagcc	aattctgagcccggagttgg
*IFN-gamma*	ctgttactgccaaggcacac	tgttaccgtccttttgccagt
*DLX1*	cctacgtccccagctacacg	gaagcgggtgagtgcgaa
*DLX2*	catgggctcctaccagtacca	cgtaggaagtgtacgcggc
*DLX5*	cggccaccgattctgactac	ctggtatgggttgagcgctt
*DLX6*	gggaaatcaggttcaacgga	agtctgctgaaagcggtggt
*Tubb3*	ggcctttggacacctattcag	tctcacattctttcctcacgac
*DCX*	cagtcagctctcaacacctaag	catctttcacatggaatcgcc
*GAD1*	gctcccagggaattagcctc	acagctctagcagggtggta
*GAD2*	ctccaacatgtacgccatgc	ctgacgtgaatgcgatgagc
